# Supplementary material for: Do factors across the World Health Organisation's International Classification of Functioning, Disability and Health framework relate to caregiver availability for community-dwelling older adults in Ghana?
Source: PLoS One. 2020 May 29;15(5):e0233541. doi: 10.1371/journal.pone.0233541 (PMC7259767; doi:10.1371/journal.pone.0233541)
Supplement: S1 Appendix — (DOCX) [file pone.0233541.s001.docx]

You are kindly invited to take part in the survey exploring the disability as well as availability of care and support for older people. This study is entirely academic and therefore your responses will only be used for academic purposes. Your response will be treated confidential and will not be revealed to a third party. Your participation in this study will be highly valued.

**Section [1]: Socio-Demographic Characteristics**

**1. Date of Birth** ……………………….…………… 2. **Sex** male female

**3. Marital status** never married currently married / cohabiting separated / divorced widowed

**4. Highest level of education completed**

no education less than primary school primary school Secondary/high school

college/pre-University postgraduate degree

**5. Ethnic background** ………………………………………………………………………………………………………….

**6. Religious denomination**

None Christianity (including roman-catholic, protestant, orthodox, other)

Islam Traditional religion Other …………………………………………………….

**7. Area of residence** Rural Urban

**8. Living arrangements**

Alone With husband/wife With children With husband/wife and children

In extended family house Other ………….………………………

**9. Employment status** Currently working Currently not working

**Section [2]: Questions about admission to hospital**

**1. What illness did the doctor / nurse diagnose you with** ………………………………………………………………………………………

**2. Were you aware of your health condition before being admitted**? Yes No

**3. Who brought you to the hospital?**

Spouse Own child A family relative A friend

Government official Other ……………………………………………………………………..

**4. How long have you been in hospital?**

Less than 1 day Less than 1 month Between 1 month and 6 months More than 6 months

**Section [3]: Functioning assessment**

These next questions ask about difficulties due to health conditions. Health conditions include diseases or illnesses, other health problems that may be short or long lasting, injuries, mental or emotional problems, and problems with alcohol or drugs.

Think back over the last 30 days, and answer these questions thinking about how much difficulty you had doing the following activities. Some of these questions may seem repetitive, but we do need your attention and it is important to give us answers to each question.

| **In the last 30 days, how much difficulty did you have…** | **None** | **Mild** | **Moderate** | **Severe** | **Extreme/**  **cannot do** | **N/A** |
| --- | --- | --- | --- | --- | --- | --- |
| 1. ...in sitting for long periods? | 1 | 2 | 3 | 4 | 5 | 9 |
| 1. …in walking 100meters? | 1 | 2 | 3 | 4 | 5 | 9 |
| 1. …in standing up from sitting down? | 1 | 2 | 3 | 4 | 5 | 9 |
| 1. …in standing for long periods? | 1 | 2 | 3 | 4 | 5 | 9 |
| 1. …with climbing one flight of stairs without resting? | 1 | 2 | 3 | 4 | 5 | 9 |
| 1. …with stooping, kneeling or crouching? | 1 | 2 | 3 | 4 | 5 | 9 |
| 1. …picking up things with your fingers (such as picking up a coin from a table)? | 1 | 2 | 3 | 4 | 5 | 9 |
| 1. ...in taking care of your household responsibilities | 1 | 2 | 3 | 4 | 5 | 9 |
| **In the last 30 days, how much difficulty did you have…** | **None** | **Mild** | **Moderate** | **Severe** | **Extreme/**  **cannot do** | **N/A** |
| 1. …in joining in community activities (for example, festivities, religious or other activities) in the same way as anyone else can? | 1 | 2 | 3 | 4 | 5 | 9 |
| 1. ...in extending your arms above shoulder level? | 1 | 2 | 3 | 4 | 5 | 9 |
| 1. …concentrating on doing something for 10 minutes? | 1 | 2 | 3 | 4 | 5 | 9 |
| 1. ...in walking a long distance such as a kilometre? | 1 | 2 | 3 | 4 | 5 | 9 |
| 1. …in bathing/washing your whole body? | 1 | 2 | 3 | 4 | 5 | 9 |
| 1. …in getting dressed? | 1 | 2 | 3 | 4 | 5 | 9 |
| 1. …in your day to day work? | 1 | 2 | 3 | 4 | 5 | 9 |
| 1. …with carrying things? | 1 | 2 | 3 | 4 | 5 | 9 |
| 1. …with moving around inside your home (such as walking across a room)? | 1 | 2 | 3 | 4 | 5 | 9 |
| 1. …with eating (including cutting up your food)? | 1 | 2 | 3 | 4 | 5 | 9 |
| 1. …with getting up from lying down? | 1 | 2 | 3 | 4 | 5 | 9 |
| 1. …with getting to and using the toilet? | 1 | 2 | 3 | 4 | 5 | 9 |
| 1. …with getting where you want to go, using private or public transport if needed? | 1 | 2 | 3 | 4 | 5 | 9 |
| 1. …getting out of your home? | 1 | 2 | 3 | 4 | 5 | 9 |
| 1. In the last 30 days, how much have you been emotionally affected by your health condition (s)? | 1 | 2 | 3 | 4 | 5 | 9 |
| 1. Overall, how much did these difficulties interfere with your life? | 1 | 2 | 3 | 4 | 5 | 9 |

**Section [4]: Support and care availability, and proximity of carers to older people**

This section will enquire about the availability of caregiver, and other features of the caregiver that can affect the support you receive.

**1. Do you regularly need help with daily tasks because of long-term illness, disability or frailty (e.g. personal care, getting around, preparing meals, etc.)?** No Yes - If ‘yes’ continue from question 3.

**2. If no, why do you not need care?**

I try to do all things by myself so that I do not become very weak, even though I am not well (ill, disability or frailty)

I do not need any help because I am healthy and strong

3. **Do you have a carer?**  1 Yes 2 No if ***NO*** continue from question 5

4. **What is your relationship with the carer?**

1 Spouse 2 Child (daughter) 3 Child (son) 4 Son/daughter-in-law

1. Unrelated family person 6 Other family member (uncle, nephew, niece)

Other, please specify ……………………….

5. **Why don’t you have a carer?**

1 My family (spouse and children) have abandoned me

2 I do not have a family (children/spouse)

3 I did not provide for my family when they were young so there are also not caring for me.

4Do not know

5 Other, please specify………….

6. **How many children do you have?**

1. None 2 1 3 2-4 4 5 or more

7. **How many of this/these (if any) provide care or support you?**

1. None 2 1 3 2-4 4 5 or more

8. **From where does the carer provide care or support you?**

1. Same home 2 From different house 3 Nearby town 4 From distant 5 N/A

9. **What is the distance (kilometres) from carer’s residence?**

1 No distance, lives in the same house 2 Less than a kilometre 3 More than a kilometre

1. N/A

10. **Do you expect your family (spouse/ or children) to care for you?**

1Yes 2 No

**Section [5]: Availability, type and frequency of care and support older people receive from all sources**

*At this point, I will like to know the kind of care you receive from your caregiver, the frequency and your satisfaction level*.

**Section [6]: Older People Relationship with Family and Friends**

At this point, I will like you to tell me the nature of your relationship with your family and friends.

*Please mark one on each line for questions 1 to 3 below.*

|  | None | 1 | 2 | 3 | 4 | 5 | 6 | 7 or more |
| --- | --- | --- | --- | --- | --- | --- | --- | --- |
| 1. How many times during the past week did you spend time with someone who does not live with you, that is, you went to see them or they came to visit you or you went out together |  |  |  |  |  |  |  |  |
| 1. How many times did you talk to someone (friends, relatives or others on the telephone in the past week (either they called you, or you called them)? |  |  |  |  |  |  |  |  |
| 1. About how often did you go to meetings of clubs, religious meetings or other groups that you belong to in the past week? |  |  |  |  |  |  |  |  |

*Please select one on each line for 1 to 6 below*

|  | Hardly ever | Some of the time | Most of the time |
| --- | --- | --- | --- |
| 1. Does it seem that your family and friends (people who are important to you) understand you? |  |  |  |
| 1. Do you feel useful to your family and friends (people important to you)? |  |  |  |
| 1. Do you know what is going on with your family and friends? |  |  |  |
| 1. When you are talking with your family and friends, do you feel you are being listened to? |  |  |  |
| 1. Do you feel you have a definite role (place) in your family and among your friends? |  |  |  |
| 1. Can you talk about your deepest problems with at least some of your family and friends? |  |  |  |

**Section [7]: Kind of support from sources**

1. **Do you receive support from other sources?**

1 Yes 2 No if **NO continue from** **Section 8**

1. **If yes, where do you receive support? (mark all those that apply)**

|  | Yes | No | Do not know |
| --- | --- | --- | --- |
| Neighbours/community |  |  |  |
| Government |  |  |  |
| Religious group/members (church, etc) |  |  |  |
| Non-governmental Organisation |  |  |  |
| Other…………………………… |  |  |  |
