# Supplementary material for: Do factors across the World Health Organisation's International Classification of Functioning, Disability and Health framework relate to caregiver availability for community-dwelling older adults in Ghana?
Source: PLoS One. 2020 May 29;15(5):e0233541. doi: 10.1371/journal.pone.0233541 (PMC7259767; doi:10.1371/journal.pone.0233541)
Supplement: S1 Table — (DOCX) [file pone.0233541.s002.docx]

**S1 Table: Bivariate analysis of perceived caregiver availability across WHO-ICF components**

| Demographic Characteristics (N=400) | Total N (%) | Perceived caregiver availability | | p-value |
| --- | --- | --- | --- | --- |
| PERSONAL FACTORS |  | **Yes N (%)** | **No N (%)** |  |
| Age *(mean, SD)* | 71.3±8.42 | 71.6±8.56 | 69.3±7.31 | 0.067 |
| Sex |  |  |  | 0.899 |
| Male | 196 (49.0) | 169 (49.1) | 27 (48.2) |  |
| Female | 204 (51.0) | 175 (50.9) | 29 (51.8) |  |
| Marital status |  |  |  | 0.019 |
| Single/separated/divorced | 58 (14.5) | 46 (13.4) | 12 (21.4) |  |
| Currently married/cohabiting | 212 (53.0) | 192 (55.8) | 20 (35.7) |  |
| Widowed | 130 (32.5) | 106 (30.8) | 24 (42.9) |  |
| Education |  |  |  | 0.334 |
| No education | 128 (32.0) | 106 (30.8) | 22 (39.3) |  |
| At least junior high completed | 209 (52.3) | 181 (52.6) | 28 (50.0) |  |
| At least senior high completed | 63 (15.8) | 57 (16.6) | 6 (10.7) |  |
| Religion |  |  |  | 0.140 |
| None | 27 (6.75) | 20 (5.81) | 7 (12.5) |  |
| Christianity | 331 (82.8) | 286 (83.1) | 45 (80.4) |  |
| Islam | 42 (10.0) | 38 (11.1) | 4 (7.14) |  |
| Residence |  |  |  | 0.723 |
| Rural | 227 (56.8) | 194 (56.4) | 33 (58.9) |  |
| Urban | 173 (43.3) | 150 (43.6) | 23 (41.1) |  |
| Living arrangement |  |  |  | 0.002 |
| Alone | 50 (12.5) | 35 (10.2) | 15 (26.8) |  |
| With couple | 100 (25.0) | 89 (25.9) | 11 (19.6) |  |
| With couple and children | 250 (62.5) | 220 (64.0) | 30 (53.6) |  |
| Employment status |  |  |  | 0.351 |
| Currently working | 156 (39.0) | 131 (38.1) | 25 (44.6) |  |
| Currently not working | 244 (61.0) | 213 (61.9) | 31 (55.4) |  |
| BODY FUNCTION AND STRUCTURE |  |  |  |  |
| Injury |  |  |  | 0.976 |
| Yes | 135 (33.8) | 116 (33.7) | 19 (33.9) |  |
| No | 265 (66.3) | 228 (66.3) | 37 (66.1) |  |
| CHRONIC HEALTH CONDITION |  |  |  |  |
| Multi-morbidity |  |  |  | **0.009** |
| No condition | 135 (33.8) | 117 (34.0) | 18 (32.1) |  |
| Any 1 condition | 173 (43.3) | 140 (40.7) | 33 (58.9) |  |
| Any 2 or more conditions | 92 (23.0) | 87 (25.3) | 5 (8.93) |  |
| ACTIVITY LIMITATION |  |  |  |  |
| Disability score (mean, SD) | 56.9±22.0 | 56.5±22.6 | 59.0±18.7 | 0.430 |
|  |  |  |  |  |
| ENVIRONMENTAL FACTORS |  |  |  |  |
| *Perceived Support* |  |  |  |  |
| Family and friend understand you |  |  |  | <0.001 |
| Hardly ever | 85 (21.3) | 59 (17.2) | 26 (46.4) |  |
| Some of the time | 162 (40.5) | 149 (43.3) | 13 (23.2) |  |
| Most of the time | 153 (38.3) | 136 (39.5) | 17 (30.4) |  |
| Feel useful to family and friends |  |  |  | <0.001 |
| Hardly ever | 83 (20.8) | 58 (16.9) | 25 (44.6) |  |
| Some of the time | 173 (43.3) | 156 (45.4) | 17 (30.4) |  |
| Most of the time | 144 (36.0) | 130 (37.8) | 14 (25.0) |  |
| Awareness of matters concerning family and friends |  |  |  | 0.019 |
| Hardly ever | 179 (44.8) | 145 (42.2) | 34 (60.7) |  |
| Some of the time | 112 (28.0) | 98 (28.5) | 14 (25.0) |  |
| Most of the time | 109 (27.3) | 101 (29.4) | 8 (14.3) |  |
| Being listened to by family and friends |  |  |  | <0.001 |
| Hardly ever | 103 (25.8) | 75 (21.8) | 28 (50.0) |  |
| Some of the time | 167 (41.8) | 150 (43.6) | 17 (30.4) |  |
| Most of the time | 130 (32.5) | 119 (34.6) | 11 (19.6) |  |
| Have a definite role towards family and friends |  |  |  | <0.001 |
| Hardly ever | 90 (22.5) | 67 (19.5) | 23 (41.1) |  |
| Some of the time | 162 (40.5) | 139 (40.4) | 23 (41.1) |  |
| Most of the time | 148 (37.0) | 138 (40.1) | 10 (17.9) |  |
| Share deepest problems with some family and friends |  |  |  | <0.001 |
| Hardly ever | 83 (20.8) | 61 (17.7) | 22 (39.3) |  |
| Some of the time | 173 (43.3) | 149 (43.3) | 24 (42.9) |  |
| Most of the time | 144 (36.0) | 134 (39.0) | 10 (17.9) |  |
| *Emotional support* |  |  |  |  |
| Often time you spoke with someone via telephone (past week) |  |  |  | 0.788 |
| None | 173 (43.3) | 150 (43.6) | 21 (41.1) |  |
| 1-5 times | 161 (40.3) | 139 (40.4) | 22 (39.3) |  |
| 6 or more times | 66 (16.5) | 55 (16.0) | 11 (19.6) |  |
| Spent time with someone who does not live with you (past week) |  |  |  | <0.01 |
| None | 54 (13.5) | 38 (11.1) | 16 (28.6) |  |
| 1-5 times | 196 (49.0) | 172 (50.0) | 24 (42.9) |  |
| 6 or more times | 150 (37.5) | 134 (39.0) | 16 (28.6) |  |
| Neighbours/community support |  |  |  | <0.001 |
| Yes | 222 (55.5) | 203 (59.0) | 19 (33.9) |  |
| No | 178 (44.5) | 141 (41.0) | 37 (66.1) |  |
| Government support |  |  |  | 0.329 |
| Yes | 174 (43.5) | 153 (44.5) | 21 (37.5) |  |
| No | 226 (56.5) | 191 (55.5) | 35 (62.5) |  |
| Religious group/members support |  |  |  | 0.009 |
| Yes | 235 (58.8) | 165 (41.3) | 24 (42.9) |  |
| No | 165 (41.3) | 133 (38.7) | 32 (57.1) |  |
| Non-government organisation support |  |  |  | 0.196 |
| Yes | 10 (2.50) | 10 (2.91) | 0 (0.00) |  |
| No | 390 (97.5) | 334 (97.1) | 56 (100) |  |
| Number of children |  |  |  | 0.009 |
| At most one child | 40 (10.0) | 31 (9.01) | 9 (16.1) |  |
| 2-4 | 128 (32.0) | 102 (29.7) | 26 (46.4) |  |
| 5 or more | 232 (58.0) | 211 (61.3) | 21 (37.5 |  |
| PARTICIPATION |  |  |  |  |
| Often times you attend meetings (past week) |  |  |  | 0.966 |
| None | 223 (95.7) | 223 (95.7) | 46 (95.8) |  |
| At least once | 12 (4.27) | 10 (4.29) | 2 (4.17) |  |
