# Supplementary material for: Do factors across the World Health Organisation's International Classification of Functioning, Disability and Health framework relate to caregiver availability for community-dwelling older adults in Ghana?
Source: PLoS One. 2020 May 29;15(5):e0233541. doi: 10.1371/journal.pone.0233541 (PMC7259767; doi:10.1371/journal.pone.0233541)
Supplement: S2 Table — (DOCX) [file pone.0233541.s003.docx]

**S2 Table: Full Model for perceived caregiver availability according to WHO-ICF frameworks**

| Variables based on ICF component | COR | Model 1  (PF)-AOR | Model 2  (PF+BFS)- AOR | Model 3  (PF+BFS+HC)- AOR | Model 4  (PF+BFS+HC+AL)- AOR | Model 5  PF+BFS+HC+AL + PR)- AOR | Model 6  PF+BFS+HC+AL+PR+EF)- AOR |
| --- | --- | --- | --- | --- | --- | --- | --- |
| PERSONAL FACTORS |  |  |  |  |  |  |  |
| Age *(mean, SD)* | 1.04 (1.00, 1.07)* | 1.04 (1.00, 1.08)* | 1.04 (1.00, 1.08)* | 1.03 (0..99, 1.07)* | 1.03 (0..99, 1.07)* | 1.03 (0..99, 1.07)* | 1.03 (0.99, 1.08)* |
| Gender |  |  |  |  |  |  |  |
| Male (vs female) | 1.04 (0.59, 1.83) |  |  |  |  |  |  |
| Marital status |  |  |  |  |  |  |  |
| Single/separated/divorced (vs married/cohabiting) | 0.40 (0.18, 0.88)*** | 0.50 (0.22, 1.13)* | 0.50 (0.22, 1.13)* | 0.47 (0.21, 1.05)* | 0.47 (0.21, 1.05)* | 0.47 (0.21, 1.05)* | 0.67 (0.27, 1.68) |
| Widowed (vs married/cohabiting) | 0.46 (0.24, 0.87)** | 0.45 (0.23, 0.87)** | 0.45 (0.23, 0.87)** | 0.48 (0.25, 0.93)** | 0.48 (0.25, 0.93)** | 0.48 (0.25, 0.93)** | 0.49  (0.24, 1.00)** |
| Education |  |  |  |  |  |  |  |
| No education (vs at most junior high completed) | 0.75 (0.41, 1.37) |  |  |  |  |  |  |
| At least senior high completed (vs at most junior high completed) | 1.47 (0.58, 3.73) |  |  |  |  |  |  |
| Religion |  |  |  |  |  |  |  |
| None (vs Christianity) | 0.45 (0.18, 1.12)* | 0.52 (0.19, 1.41) |  |  |  |  |  |
| Islam (vs Christianity) | 1.50 (0.51, 4.39) | 1.12 (0.37, 3.40) |  |  |  |  |  |
| Residence |  |  |  |  |  |  |  |
| Urban (vs rural) | 1.11 (0.63, 1.97) |  |  |  |  |  |  |
| Living arrangement |  |  |  |  |  |  |  |
| Alone (vs With couple and children) | 0.32 (0.16, 0.65)*** | 0.42 (0.19, 0.89)** | 0.42 (0.19, 0.89)** | 0.37 (0.17, 0.77)*** | 0.37 (0.17, 0.77)*** | 0.37 (0.17, 0.77)*** | 0.83 (0.34, 1.99) |
| With couple (vs With couple and children) | 1.10 (0.53, 2.30) | 1.10 (0.0.52, 2.33) | 1.10 (0.0.52, 2.33) | 1.03 (0.48, 2.21) | 1.03 (0.48, 2.21) | 1.03 (0.48, 2.21) | 1.08 (0.48, 2.43) |
| Employment status |  |  |  |  |  |  |  |
| Currently working (vs Currently not working) | 0.76 (0.43, 1.35) |  |  |  |  |  |  |
| BODY FUNCTION AND STRUCTURE |  |  |  |  |  |  |  |
| Visual impairment |  |  |  |  |  |  |  |
| Yes (vs no) | 0.65 (0.07, 5.90) |  |  |  |  |  |  |
| Injury |  |  |  |  |  |  |  |
| Yes (vs no) | 1.38 (0.52, 3.66) |  |  |  |  |  |  |
| HEALTH CONDITION |  |  |  |  |  |  |  |
| Multi-morbidity |  |  |  |  |  |  |  |
| No chronic condition (vs any 1 condition) | 1.53 (0.82, 2.86) |  |  | 1.60 (0.84, 3.06)* | 1.60 (0.84, 3.06)* | 1.60 (0.84, 3.06)* | 1.67 (0.83, 3.68)* |
| Any 2 or more chronic conditions (vs any 1 condition) | 4.10 (1.54, 10.9)*** |  |  | 3.90 (1.43, 10.6)*** | 3.90 (1.43, 10.6)*** | 3.90 (1.43, 10.6)*** | 3.58 (1.24, 10.3)** |
| ACTIVITY LIMITATION |  |  |  |  |  |  |  |
| Disability score (mean, SD) | 1.00 (0.98, 1.00) |  |  |  |  |  |  |
| PARTICIPATION RESTRICTION |  |  |  |  |  |  |  |
| Often times you attend meetings (past week) |  |  |  |  |  |  |  |
| At least once (vs None) | 1.03 (0.22, 4.86) |  |  |  |  |  |  |
| ENVIRONMENTAL FACTORS |  |  |  |  |  |  |  |
| *Perceived Support* |  |  |  |  |  |  |  |
| Family and friend understand you |  |  |  |  |  |  |  |
| Hardly ever (vs some of the time) | 0.20 (0.10, 0.41)*** |  |  |  |  |  | 0.19 (0.08, 0.43)**** |
| Most of the time (vs some of the time) | 0.70 (0.33, 1.49) |  |  |  |  |  | 0.69 (0.30, 1.58) |
| *Emotional support* |  |  |  |  |  |  |  |
| Spent time with someone who does not live with you (past week) |  |  |  |  |  |  |  |
| None (vs 1-5 times) | 0.33 (0.16, 0.68)*** |  |  |  |  |  | 0.38 (0.17, 0.87)** |
| 6 or more times (vs 1-5 times) | 1.17 (0.60, 2.29) |  |  |  |  |  | 0.69 (0.32, 1.49) |
| Often time you spoke with someone via telephone (past week) |  |  |  |  |  |  |  |
| 1-5 times (vs none) | 0.97 (0.52, 1.82) |  |  |  |  |  |  |
| 6 or more times (vs none) | 0.77 (0.35, 1.68) |  |  |  |  |  |  |
| Neighbours/community support |  |  |  |  |  |  |  |
| No (vs Yes) | 0.42 (0.21, 0.85)* |  |  |  |  |  | 0.39 (0.19, 0.80)*** |
| Government support |  |  |  |  |  |  |  |
| No (vs Yes) | 1.01 (0.51, 2.00) |  |  |  |  |  |  |
| Number of children |  |  |  |  |  |  |  |
| At most one child (vs 5 or more) | 0.34 (0.14, 0.82)* |  |  |  |  |  | 0.90 (0.31, 2.58) |
| 2-4 (5 or more) | 0.39 (0.21, 0.73)** |  |  |  |  |  | 0.48 (0.24, 1.00)**` |

**PF- personal factors; BFS- body function and structure; H- health condition; E- environmental factors; AL-activity limitation; P-participation; AJHS-** **At least junior high completed; WCC-** **with couple and children; Significant at *p-value < 0.2; **p-value < 0.05; ***p-value < 0.01, ****p-value<0.001**
